# Prescription Department

**Published:** 1891-09

**Authors:** 


CHOLERA MORBUS—
℞ Acid sulph. arom., dr. ij.
Ext. haematoxylon, dr. ij.
Spt. chloroformi, oz. ss.
Syr. Zingeberis, q. s. oz. iij.
M. S. Teaspoonful every two hours.

ACUTE CYSTITIS—
℞ Tinct. aconiti, dr. j.
Spirit aether nitrosi, oz, j.
Liq. rotas. citratis, q. s. oz. vj.
M. S. Dessertspoonful every four hours until all fever ceases and the pulse is quiet.

PROLAPSUS RECTI—By Q. A. Shuford, M. D., Tyler, Texas.
℞ Oint. nit. mercury, 2 drachms.
Resin ointment, 2 drachms.
Ext. opium, half drachm.
Carbolic acid, 1 drachm.
Cosmoline, 1 ounce.
M. ft. ointment.
Sig. Saturate a lump of cotton and pass it up the rectum after the bowels move and the bath in the morning, also bathe and anoint at night. I am inclined to think that this lump of cotton is of great benefit in helping t o prevent prolapsus and at the same time constantly medicating the parts.

BURNS AND SCALDS—Bartholow.
℞ Acidi sallicylici, 1 drachm,
Olei, Olivae, 8 fl. ounces.
M. Sig. Apply to burn, covering with linen or lint.—Ex.

HEART STIMULANT—By Prof. Hichard, France.
℞ Benzoate of sodium.
Caffeine, aa gr. xlv.
Ext. stigmata maidis dr. jss.
M. For 60 pills.
Sig. Two pills twice or three times a day.

OR

℞ Ext. cinchona, dr. ijss.
Benzoate of soda.
Caffeine, aa gr. lxv.
M. For 100 pills. Sig. Two pills before each meal.—Ex.

VARICOSE VEINS.—
℞ Barii chlorid, 15 grains.
Aquae distill., q. s.
Lanolin, 2 drachms.
Ol amygdalae dulc, 1 drachm.
M. Sig. Dissolve barium in water by shaking, then add fatty mixture. Rub on the dilated veins three times daily.—Ex.

The following prescriptions were taken from Hare's Therapeutics:

ACUTE BRONCHITIS—

R Syr. ipecac, f dr. j.
Potas. citrat., dr. iv.
Aq. distillat, q. s. f oz. vj.

M. Sig. Dessertspoonful every 4 hours for a child of five years.

OR

R Syr. ipecac, f oz. ij.
Succus lemonis, f oz. j.
Potas. carbonat, dr. iv.
Spirit aether nitrosi, f oz. j.
Aq. dist. q. s. f oz. vj,

M. Sig. Dessertspoonful every 4 hours for an adult.

CHOLERA INFANTUM—

R Acid sulph. aromat. gtt. xxiv.,
Tinct. opii camphorat , f dr. iij.
Elixir curacoae, f dr. ij.
Aqua cinnamoni, q. s. f dr. iij.

M Sig. Teaspoonful every two hou

OR

R Acid sulph. aromat., gtt. xxiv.
Ol. caryophy, m viij.
Tinct. opii camphorat., f dr. j.
Spirit chloroformi, gtt. xlviij.
Syr. zingeberis. q. s. f oz. iij.

M. S. As above.

CONSTIPATION—

R Aloes soc., gr. xx.
Ext. nuc vom., gr. iv.
Ext. physostig., gr. iij.
Ext. belladon., gr. iv.

M. Ft. in pil. No. xx.
Sig. One pill at night, or night and morning.

CORNS—

R Acid sallicylic, gr. xxx.
Ext. canabis indica, gr. x.
Collodii, oz. ss.

M. S. Apply with camel's hair night and morning for several days. Then soak in hot water, when the corns will readily come away.

